# The Periplasmic Protein TolB as a Potential Drug Target in *Pseudomonas aeruginosa*

**DOI:** 10.1371/journal.pone.0103784

**Published:** 2014-08-05

**Authors:** Alessandra Lo Sciuto, Regina Fernández-Piñar, Lucia Bertuccini, Francesca Iosi, Fabiana Superti, Francesco Imperi

**Affiliations:** 1 Department of Biology and Biotechnology “Charles Darwin”, Sapienza University of Rome, Rome, Italy; 2 Ultrastructural Infectious Pathology Section, Department of Technology and Health, National Institute of Health, Rome, Italy; 3 Pasteur Institute-Cenci Bolognetti Foundation, Sapienza University of Rome, Rome, Italy; Queen's University Belfast, United Kingdom

## Abstract

The Gram-negative bacterium *Pseudomonas aeruginosa* is one of the most dreaded pathogens in the hospital setting, and represents a prototype of multi-drug resistant “superbug” for which effective therapeutic options are very limited. The identification and characterization of new cellular functions that are essential for *P. aeruginosa* viability and/or virulence could drive the development of anti-*Pseudomonas* compounds with novel mechanisms of action. In this study we investigated whether TolB, the periplasmic component of the Tol-Pal trans-envelope protein complex of Gram-negative bacteria, represents a potential drug target in *P. aeruginosa*. By combining conditional mutagenesis with the analysis of specific pathogenicity-related phenotypes, we demonstrated that TolB is essential for *P. aeruginosa* growth, both in laboratory and clinical strains, and that TolB-depleted *P. aeruginosa* cells are strongly defective in cell-envelope integrity, resistance to human serum and several antibiotics, as well as in the ability to cause infection and persist in an insect model of *P. aeruginosa* infection. The essentiality of TolB for *P. aeruginosa* growth, resistance and pathogenicity highlights the potential of TolB as a novel molecular target for anti-*P. aeruginosa* drug discovery.

## Introduction

Nowadays, microbial resistance to antibiotics is a major hindrance to the successful treatment of many bacterial infections. Since resistance to a given antibiotic inevitably builds up over time, the development of new antibacterial drugs with novel mechanisms of action represents an important strategy against antibiotic resistance. Rational development of antibacterial drugs with innovative activities involves the identification of new cellular targets, that may emerge from a better understanding of cellular pathways critical for pathogen survival and/or pathogenicity.

TolB is the periplasmic component of the Tol-Pal system, a multi-protein complex present in almost all Gram-negative bacteria which connects the cytoplasmic (or inner) membrane with the outer membrane [1]. The Tol-Pal system was discovered as the protein machinery responsible for the internalization of the group A colicins and filamentous phage DNA in the model bacterium *Escherichia coli* [2], [3]. However, further studies have demonstrated that this system also plays a relevant role in the maintenance of cell envelope integrity and in the cell division process in almost all Gram-negative bacteria investigated to date [1], [4]–[9].

With the exception of *Erwinia chrysanthemi* and *Caulobacter crescentus* [10], [11], the Tol-Pal system does not appear to be essential for bacterial growth *in vitro*, as demonstrated in several Enterobacteriaceae, *Pseudomonas putida* and *Vibrio cholerae*, although Tol-Pal defective mutants generally showed increased sensitivity to toxic compounds (e.g. antibiotics) and reduced ability to cause infection [7], [8], [12]–[14]. Proteomic analysis showed that TolB is one of the most abundant proteins in the periplasm of the human pathogen *Pseudomonas aeruginosa* [15]. Notably, several attempts to generate *tolB* mutants in this bacterium, by either site-directed [16] or large-scale random transposon mutagenesis [17]–[19] failed, suggesting that *tolB* could indeed be essential in *P. aeruginosa*.

In this study, we used a conditional mutagenesis approach coupled with the analysis of specific pathogenicity-related phenotypes to verify the essentiality of the *tolB* gene in *P. aeruginosa*, and to evaluate TolB as a potential target for the development of novel anti-*P. aeruginosa* drugs.

## Materials and Methods

### Ethics statement

Human serum was obtained from five healthy volunteers who gave their written informed consent to the study. The research project was approved by the review board of the Pasteur Institute-Cenci Bolognetti Foundation, Sapienza University of Rome (Rome, Italy).

### Bacteria and growth conditions

Bacterial strains and plasmids used in this study are listed in Table 1. Bacteria were grown in Mueller-Hinton (MH) broth or M9 minimal medium with 20 mM succinate (SM9) [27], containing or not arabinose at different concentrations. When indicated, sucrose was added to the growth medium to increase osmolarity.

**Table 1:** Bacterial strains and plasmids used in this study.

Strain or plasmid	Genotype and/or relevant characteristics	Reference or source
***P. aeruginosa***		
PAO1 (ATCC15692)	Prototroph	American type culture collection
PA14	Prototroph	[20]
TR1	Prototroph; cystic fibrosis isolate	[21]
PAO1 *araC*P_BAD_ *tolB*	PAO1 with an arabinose-inducible additional copy of *tolB* inserted into the *attB* neutral site	This work
PAO1 Δ*tolB araC*P_BAD_ *tolB*	PAO1 *araC*P_BAD_ *tolB* deleted of the endogenous copy of *tolB*	This work
PA14 *araC*P_BAD_ *tolB*	PA14 with an arabinose-inducible additional copy of *tolB* inserted into the *attB* neutral site	This work
PA14 Δ*tolB araC*P_BAD_ *tolB*	PA14 *araC*P_BAD_ *tolB* deleted of the endogenous copy of *tolB*	This work
TR1 *araC*P_BAD_ *tolB*	TR1 with an arabinose-inducible additional copy of *tolB* inserted into the *attB* neutral site	This work
TR1 Δ*tolB araC*P_BAD_ *tolB*	TR1 *araC*P_BAD_ *tolB* deleted of the endogenous copy of *tolB*	This work
***E. coli***		
S17.1λ*pir*	*thi pro hsdR hsdM^+^ recA RP4-2-Tc::Mu-Km::Tn7 λpir, Gm^R^*	[22]
DH5αF'	*recA1 endA1 hsdR17 supE44 thi-1 gyrA96 relA1* Δ(*lacZYA-argF*)U169[φ*80 dlacZ*Δ*M15*], Nal^R^	[23]
**Plasmid**		
pBluescript-II KS+	Cloning vector; ColE1 replicon; Ap^R^	Stratagene
pDM4	Suicide vector; *sacBR*, *oriR6K*; Cm^R^	[23]
pDM4Δ*tolB*	pDM4 derivative for *tolB* in-frame deletion; Cm^R^	This work
pBEM9	Vector carrying the *araC*P_BAD_ regulatory region with an altered RBS for stringent arabinose-dependent control in *P. aeruginosa*; Ap^R^	[24]
pBEM9-*tolB*	pBEM9 derivative carrying the *tolB* coding sequence cloned by HindIII/EcoRI digestion downstream of the *araC*P_BAD_ regulatory region	This work
mini-CTX1	Self-proficient integration vector with *tet,* Ω-*FRT*-*attP*-MCS, *ori, int,* and *oriT*; Tc^R^	[26]
mini-CTX1-*araC*P_BAD_ *tolB*	mini-CTX1 derivative carrying the *araC*P_BAD_ *tolB* from pBEM9 cloned by XhoI/EcoRI digestion	This work

### Construction of *tolB* conditional mutants

Primers and restriction enzymes used for cloning are listed in Table S1. Plasmid mini-CTX1-*araC*P_BAD_ *tolB* was generated by cloning the *tolB* coding sequence into pBEM9 downstream to an *araC*-P_BAD_ regulatory region that was optimized for *P. aeruginosa* by modification of the ribosome binding site [25], followed by subcloning of the entire *araC*P_BAD_ *tolB* region into the integration-proficient vector mini-CTX1 [26]. The mini-CTX1-*araC*P_BAD_ *tolB* construct was integrated into the *attB* neutral site of the *P. aeruginosa* chromosome, and the backbone plasmid removed as described [28]. In-frame deletion of the endogenous *tolB* copy was obtained using the *sacB*-based suicide vector pDM4 as previously described [24]. All constructs were verified by DNA sequencing.

### Detergent, serum and antibiotic sensitivity assays

Sensitivity to the lytic effect of SDS was assessed by determining the turbidity (OD_600_) of bacterial cell suspensions in saline after 5-min incubation at room temperature in the presence of increasing SDS concentrations (0–5%). Serum sensitivity was determined by incubating about 10^8^ *P. aeruginosa* cells at 37°C in saline in the presence of 50% human serum (pooled from five healthy volunteers) or heat-inactivated human serum [29]. Ofloxacin sensitivity was determined by incubating about 10^8^ *P. aeruginosa* colony-forming units (CFUs) at 37°C in saline in the presence or in the absence of 0.5 mg/L ofloxacin, corresponding to the minimum inhibitory concentration (MIC) for the PAO1 strain [30] (data not shown). After 3 h, ten-fold serial dilutions of each cell suspension were plated on MH agar with 0.2% arabinose to determine the percentage of survival with respect to the corresponding controls. Sensitivity to polymyxin B and colistin was assessed by a modification of a previously-described assay [31]. Briefly, about 10^6^ *P. aeruginosa* CFUs were incubated at 37°C in saline containing 4, 1 or 0.25 mg/L colistin or 2, 0.5 or 0.125 mg/L polymyxin B, corresponding to 4×, 1× or 0.25× MICs for the PAO1 wild-type strain, respectively (data not shown). After 1 h, ten-fold serial dilutions of each cell suspension were plated as described above to determine percentage of survival with respect to untreated controls. Resistance to the growth inhibitory activity of several antibiotics was assessed by the Kirby-Bauer disc diffusion test in MH agar supplemented or not with 0.01 or 0.005% arabinose, using disks containing gentamicin (10 µg), streptomycin (10 µg), tetracycline (30 µg), ampicillin (10 µg), ciprofloxacin (5 µg), imipenem (10 µg), ceftazidime (30 µg), colistin (10 µg) (Becton Dickinson), or polymyxin B (300 units; Oxoid). Growth inhibition halo diameters were measured after 20 or 40 h of growth at 37°C for PAO1 or the PAO1 *tolB* conditional mutant, respectively.

### *Galleria mellonella* infection and persistence assays

*P. aeruginosa* strains were grown in MH with 0.2% arabinose, and serial dilutions of bacterial cell suspensions in saline were injected into *G. mellonella* larvae as described [32]. Larvae were incubated at 30°C for one week to monitor mortality. The lethal dose 90% (LD_90_) was determined as described [29]. *P. aeruginosa* persistence in *G. mellonella* larvae was assessed by infecting larvae with about 10^6^ CFUs. After 2 h of incubation at 30°C, larvae were cut with a razor blade to recover the hemolymph. Ten-fold serial dilutions of the hemolymph were plated on Pseudomonas Isolation Agar containing 0.2% arabinose to determine the percentage of viable cells with respect to the initial inoculum (infecting dose).

### Electron microscopy

Scanning (SEM) and transmission electron microscopy (TEM) were performed using previously described procedures [33]. For SEM examination, bacterial cells were fixed with 2.5% glutaraldehyde in 0.1 M sodium cacodylate buffer (pH 7.4) overnight at 4°C, seeded onto polylisinated glass coverslips, left to adhere for 2 h at room temperature, and postfixed with 1% OsO_4_ in 0.1 M sodium cacodylate buffer for 1 h at room temperature. Samples were then dehydrated through a graded series of ethanol solutions, critical point dried and gold sputtered, and examined with a SEM Inspect F (FEI) scanning electron microscope. For TEM analysis, bacterial cells were fixed with 2.5% glutaraldehyde, 2% paraformaldehyde and 2 mM CaCl_2_ in 0.1 M sodium cacodylate buffer (pH 7.4) overnight at 4°C. After incubation, cells were washed in cacodylate buffer and postfixed with 1% OsO_4_ in 0.1 M sodium cacodylate buffer for 1 h at room temperature, treated with 1% tannic acid in 0.05 M cacodylate buffer for 30 min and rinsed in 1% sodium sulphate in 0.05 cacodylate for 10 min. Fixed specimens were washed, dehydrated through a graded series of ethanol solutions (30 to 100% ethanol, each for 20 min) and embedded in Agar 100 (Agar Scientific Ltd., U.K.) (1/3 resin for 1 h and 30 min; 1/2 resin for 3 h; 2/3 resin overnight). Ultrathin sections obtained with a MT-2B Ultramicrotome (LKB – Pharmacia) were stained for 20 min with uranyl acetate (3% in 70% ethanol) and Reynold's lead citrate, and examined with an EM 208 FEI transmission electron microscope.

### Statistical analysis

Statistical analysis was performed with the software GraphPad Instat, using one-way analysis of variance (ANOVA) followed by Tukey-Kramer multiple comparison tests.

## Results and Discussion

**Figure 1:**
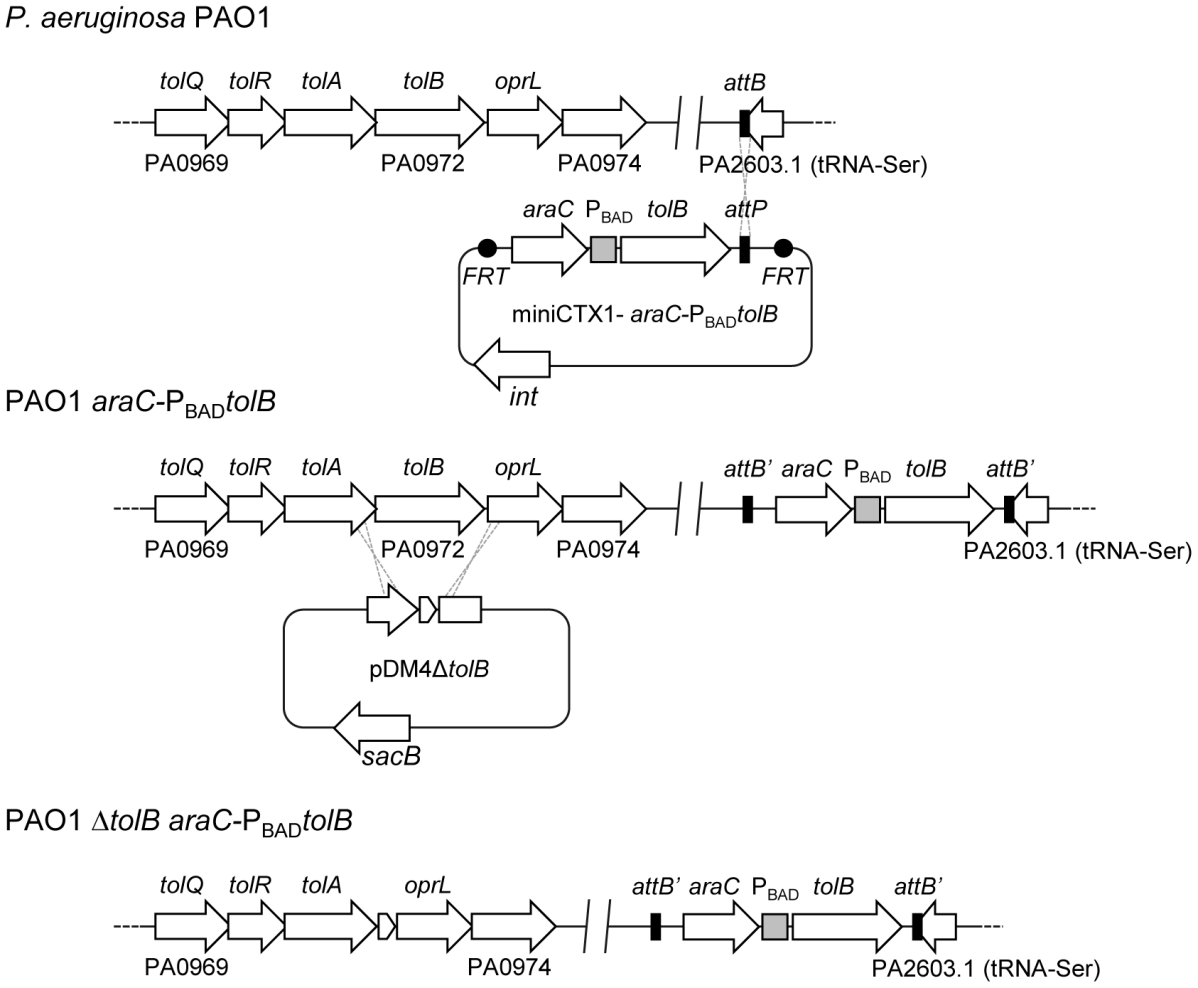
Scheme of the strategy used to generate the *P. aeruginosa* PAO1 *tolB* conditional mutant. An exogenous copy of the *tolB* coding sequence under the control of an arabinose-dependent promoter was inserted into the *attB* neutral site of the *P. aeruginosa* chromosome, by using the integration-proficient plasmid mini-CTX1-*araC*P_BAD_ *tolB* (Table 1). After Flp-mediated removal of the mini-CTX1 backbone (not shown), the resulting strain (PAO1 *araC*P_BAD_ *tolB*) is a merodiploid for *tolB*. In-frame deletion of the endogenous copy of *tolB* was obtained using the suicide plasmid pDM4Δ*tolB* (Table 1). Sucrose selection was carried out in the presence of arabinose to select removal of the pDM4 backbone, followed by PCR screening to identify clones carrying the *tolB* in-frame deletion. One of these clones was selected and used for the following analyses. This conditional mutant was named PAO1 Δ*tolB araC*P_BAD_ *tolB*.

In order to generate a stable and unmarked *P. aeruginosa tolB* conditional mutant, an arabinose-inducible copy of the *tolB* coding sequence was inserted, together with the *araC* regulatory gene, into the *attB* neutral site of the *P. aeruginosa* PAO1 chromosome. Then, in-frame deletion mutagenesis was carried out in the presence of 0.2% arabinose to remove the endogenous copy of *tolB*, leading to the generation of the *tolB* conditional mutant named PAO1 Δ*tolB araC-*P_BAD_ *tolB* (Fig. 1).

**Figure 2:**
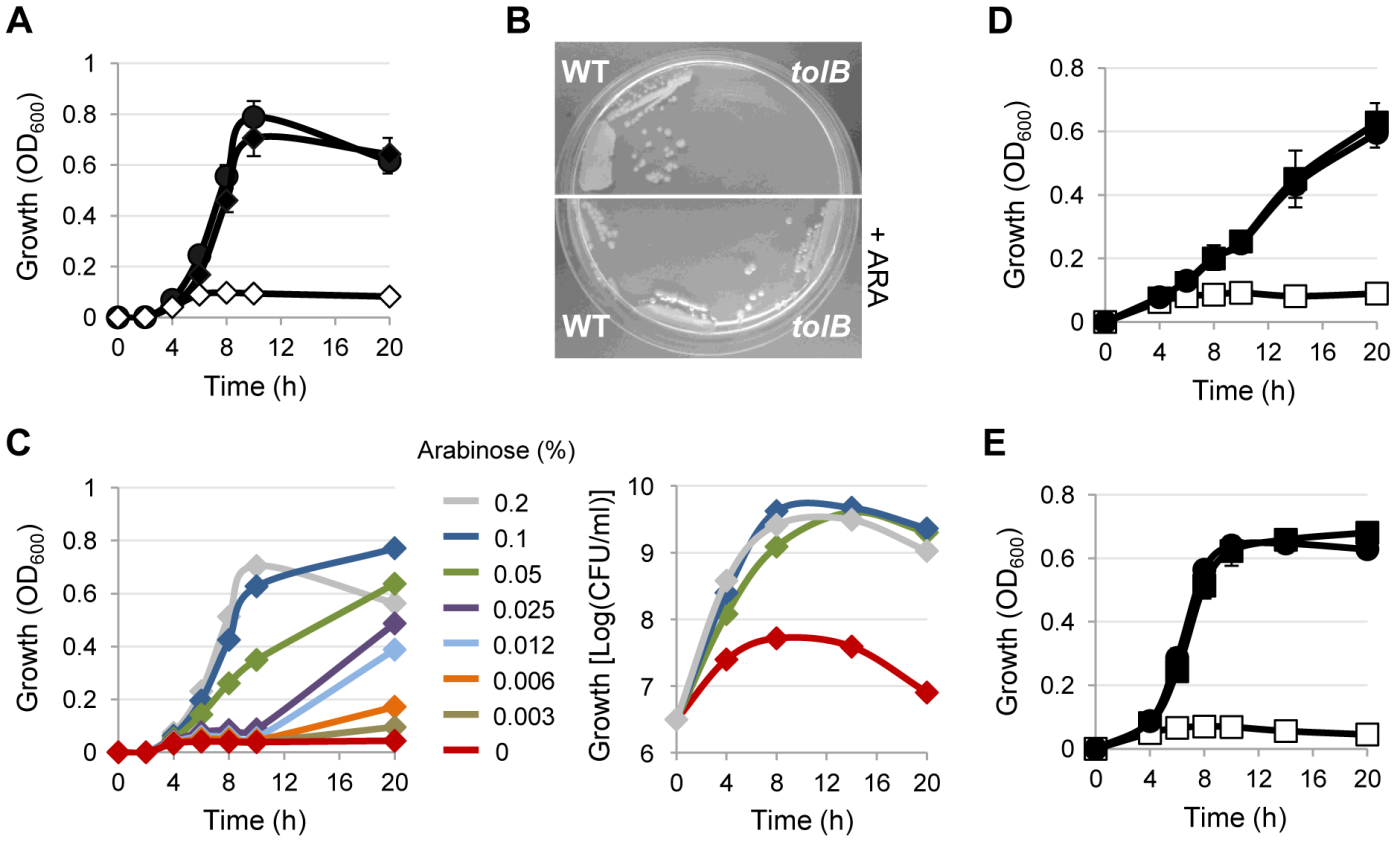
TolB is essential for *P. aeruginosa* growth *in vitro*. (A) Growth curves of the wild-type strain PAO1 (filled circles) and the PAO1 *tolB* conditional mutant in the presence (filled diamonds) or in the absence (open diamonds) of 0.2% arabinose in MH broth at 37°C in microtiter plates at 200 rpm. Results are the mean (± SD) of three independent experiments performed in triplicate. (B) Growth of PAO1 and the PAO1 *tolB* conditional mutant on MH agar plates with or without 0.2% arabinose (ARA) at 16 h. (C) Growth of the PAO1 *tolB* conditional mutant as described in legend to panel A in the presence of increasing concentrations of arabinose (0–0.2%), measured as OD_600_ (left panel) or CFU/ml (right panel). The graphs are representative of at least two independent experiments giving similar results. (D) Growth curves of *P. aeruginosa* PA14 or (E) the clinical strain TR1 (filled circles) and their corresponding *tolB* conditional mutants in the presence (filled squares) or in the absence (open squares) of 0.2% arabinose in MH broth at 37°C in microtiter plates at 200 rpm. Results are the mean (± SD) of two independent experiments performed in triplicate.

Growth of PAO1 Δ*tolB araC-*P_BAD_ *tolB* in MH in microtiter plates was almost completely abrogated unless arabinose was added to the growth medium (Fig. 2A), and the same was observed on MH agar plates (Fig. 2B). Moreover, growth of the PAO1 *tolB* conditional mutant in MH broth was proportional to the concentration of arabinose in the medium (Fig. 2C), confirming that TolB expression is tightly regulated by arabinose in the PAO1 *tolB* conditional mutant. Comparable results were obtained in SM9 minimal medium (data not shown). These data indicate that *tolB* is essential for *P. aeruginosa* PAO1 growth under laboratory conditions, and confirm the suitability of the strategy used to generate stable conditional mutants in *P. aeruginosa*.

We then verified whether the crucial role of TolB in *P. aeruginosa* growth is conserved in different genetic backgrounds. To this aim, the *tolB* conditional mutation was introduced in the reference strain PA14 and in the clinical strain TR1, isolated from a cystic fibrosis patient [21] (Table 1). As previously observed for PAO1, the growth of both PA14 and TR1 *tolB* conditional mutants was strictly dependent on the addition of arabinose to the culture medium (Figs. 2D–E), strongly suggesting that the essentiality of TolB is a conserved trait in *P. aeruginosa*.

**Figure 3:**
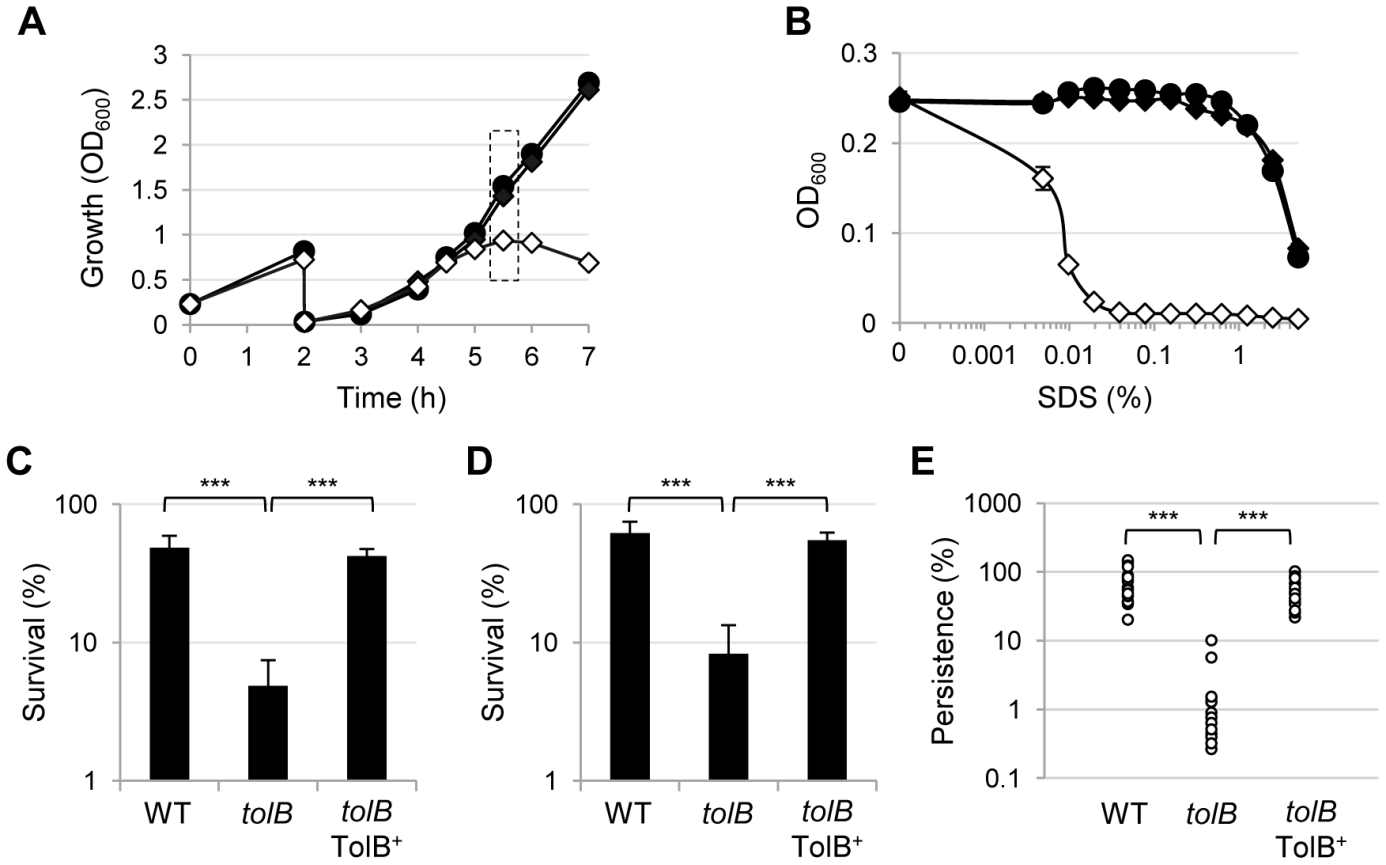
TolB is crucial for *P. aeruginosa* resistance and persistence. (A) Growth of PAO1 (circles) and PAO1 *tolB* conditional mutant (diamonds) at 37°C in MH broth at 200 rpm in flasks after two successive subcultures in the presence (filled symbols) or in the absence (open symbols) of 0.2% arabinose. The graph is representative of several assays giving similar results. (B) Lytic effect of SDS (0–5%), measured as decrease in cell suspension turbidity (OD_600_), on PAO1 wild-type cells (WT, filled circles), TolB-deficient mutant cells (*tolB*, open diamonds) and TolB-proficient mutant cells (*tolB* TolB^+^, filled diamonds). (C) Resistance of WT, *tolB* and *tolB* TolB^+^ to the bactericidal activity of 50% human serum or (D) to the bactericidal antibiotic ofloxacin (0.5 mg/L), expressed as percent survival compared to untreated cells. Results in panels B–D are the mean (± SD) of four independent experiments. (E) Persistence of WT, *tolB* and *tolB* TolB^+^ cells in *G. mellonella* larvae at 2 h post-infection. Sixteen larvae per group were infected in three independent assays. ***, P<0.001 (one-way ANOVA).

**Figure 4:**
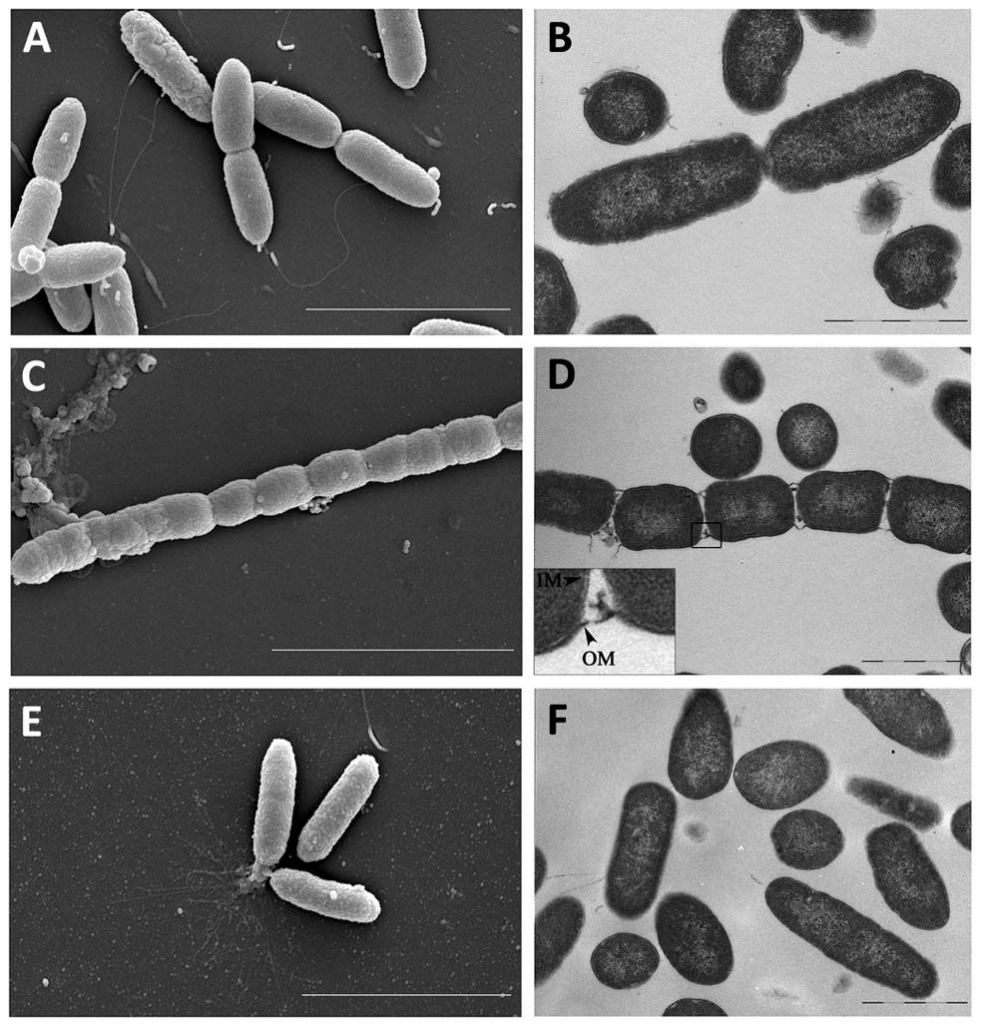
TolB-deficient cells show defects in outer membrane stability and cell division. SEM and TEM analysis (left and right panels, respectively) of PAO1 wild-type cells (A,B), TolB-deficient mutant cells (C,D) and TolB-proficient mutant cells (E,F), grown as described in the legend to Fig. 3A. Bars: 3 µm (left panels) or 1 µm (right panels). The inset in panel D shows an enlargement of the boxed area. Abbreviations: IM, inner membrane; OM, outer membrane.

In order to obtain a number of TolB-deficient cells sufficient for further analyses, a dual-refresh strategy in flask was developed, using *P. aeruginosa* PAO1 and its isogenic *tolB* conditional mutant as reference strains (Fig. 3A). Cells were grown in MH broth for 14 h in the presence of 0.2% arabinose, and then two successive refreshes were performed (starting OD_600_ of 0.25 and 0.03, respectively) in the presence or in the absence of arabinose. As soon as a growth defect was observed in the PAO1 *tolB* conditional mutant grown in the absence of arabinose (dashed box in Fig. 3A), cells were collected and tested for different phenotypes related to pathogenicity and persistence (Figs. 3B–E), as well as for cellular morphology by electron microscopy (Fig. 4).

TolB-deficient cells were almost 1,000-fold more sensitive to the detergent SDS compared to wild-type or TolB-proficient mutant cells, *i.e.* cells of the *tolB* conditional mutant grown in the presence of arabinose (Fig. 3B), suggestive of major defects in cell-envelope integrity. Accordingly, electron microscopy revealed that TolB-deficient cells form multi-septate short-cell chains, characterized by abundant generation of outer membrane blebs and release of cellular content, mainly at division sites (Fig. 4 and Figure S1), indicating that TolB deficiency in *P. aeruginosa* negatively affects cell elongation and outer membrane invagination during cell division, as previously observed in other Gram-negative bacteria [9], [34]. However, differently from what observed for the *tolB* mutant of *E. chrysanthemi* [10], growth of the *P. aeruginosa tolB* conditional mutant in the absence of arabinose could not be restored by increasing the osmolarity of the culture medium with up to 20% sucrose (Figure S2), suggesting that the growth defect of this mutant is not only related to poor cell-envelope integrity.

**Figure 5:**
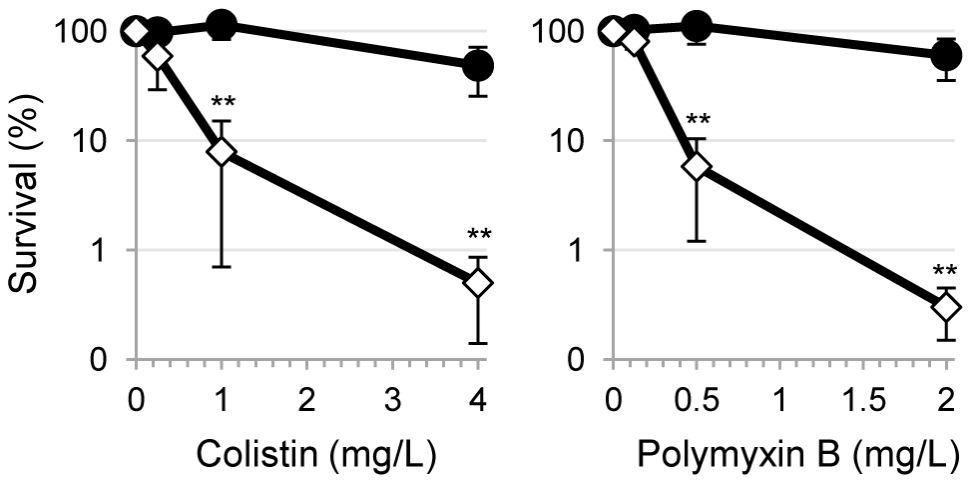
TolB depletion increases *P. aeruginosa* sensitivity to polymyxins in a killing assay. Survival of PAO1 wild-type cells (filled circles) and TolB-deficient mutant cells (open diamonds), obtained as shown in Figure 3A, after 1-h treatment with 4, 1 or 0.25 mg/L of colistin (left panel), or 2, 0.5 or 0.125 mg/L of polymyxin B (right panel). Values are expressed as percent survival compared to untreated cells, and the results represent the mean (± SD) of three independent experiments. **, P<0.01 (one-way ANOVA).

TolB-deficient cells also showed significant defects in resistance to the antibacterial activities of both human serum (Fig. 3C) and the bactericidal antibiotic ofloxacin (Fig. 3D), measured as percent survival compared to cells treated with heat-inactivated serum or untreated cells, respectively. To further investigate the contribution of TolB to antibiotic resistance, susceptibility to the growth-inhibitory activity of different antibiotics was investigated through the Kirby-Bauer disc diffusion assay, by comparing the inhibitory halos obtained by growing the PAO1 Δ*tolB araC-*P_BAD_ *tolB* conditional mutant in the presence of low (growth permissive) concentrations of arabinose with those obtained with the *P. aeruginosa* PAO1 wild type (Table 2). Cells expressing low levels of TolB showed an overall increase in sensitivity to almost all antibiotics tested, including antibiotics currently used to treat *P. aeruginosa* infections, such as the fluoroquinolone ciprofloxacin, the carbapenem imipenem and the cephalosporin ceftazidime [35], [36]. The only exceptions were ampicillin, to which *P. aeruginosa* is intrinsically insensitive due to expression of the chromosomally-encoded β-lactamase AmpC [35], [37], and polymyxins (polymyxin B and colistin), for which no significant difference in susceptibility was observed between wild-type cells and mutant cells expressing low levels of TolB (Table 2). This latter result, that was obtained by growing the *tolB* conditional mutant in the presence of low, but growth permissive concentrations of arabinose (Table 2), was verified by performing a killing assay on wild-type and TolB-depleted mutant cells, obtained through the dual-refresh strategy shown in Figure 3A. Differently from what observed with the Kirby-Bauer assay, TolB-depleted cells were significantly more sensitive to both antibiotics than wild-type cells in the killing assay (Fig. 5), indicating that the cell envelope defects associated with complete depletion of TolB (Fig. 4) can also affect resistance to polymyxins. The cationic antimicrobial peptides colistin and polymyxin B primarily act by interacting with and disrupting the outer membrane, and then damaging the cytoplasmic membrane [38]. Thus, it is plausible that, differently from other antibiotics that need to reach intracellular targets, the activity of polymyxins in the Kirby-Bauer assay (Table 2) is poorly influenced by the lower cell envelope integrity that is presumably associated with growth in the presence of reduced TolB levels.

**Table 2:** Antibiotic susceptibility by the Kirby-Bauer disk diffusion test.^a^

Strain	Arabinose (%)	Growth inhibition halo diameter (mm)								
		Gm^b^	Sm^b^	Tc^b^	Ap^b^	Ipm^b^	Caz^b^	Cip^b^	Ct^b^	PmB^b^
PAO1 (wild type)	0	21.9 ±0.8	13.6 ±1.4	12.3 ±0.7	0	25.5 ±0.6	29.3 ±1.0	32.5 ±1.9	15.5 ±0.6	13.6 ±0.7
PAO1 Δ*tolB araC-*P_BAD_ *tolB*	0.01	33.3±3.1^**^	20.0±3.5^*^	19.7±0.6^*^	0	38.8±3.0^***^	42.5±1.3^***^	43.0±2.7^***^	14.5±0.6	13.8±0.3
	0.005	36.3±3.2^***^	21.3±1.2^**^	21.3±4.2^**^	0	49.3±1.0^***^	46.3±2.1^***^	54.5±1.3^***^	15.8±0.5	14.2±0.7

a Growth inhibition halo diameters were measured after 20 (PAO1) or 40 h (PAO1 Δ*tolB araC-*P_BAD_ *tolB*) of growth at 37°C on MH agar plates, containing or not arabinose at the indicated concentration. Values are the average ±SD of at least three independent assays. Asterisks indicate statistically significant differences compared to wild type (one-way ANOVA; ^*^ *P*<0.05; ^**^ *P*<0.01; ^***^ *P*<0.001).

b Abbreviations: Gm, gentamycin; Sm; streptomycin, Tc, tetracyclin, Ap, ampicillin; Ipm, imipenem; Cip, ciprofloxacin, Caz, ceftazidime, Ct, colistin, PmB, polymyxin B.

Since laboratory cultures not always reflect bacterial growth and virulence during infection, we also assessed the ability of the PAO1 *tolB* conditional mutant to cause infection and persist in the well-established *G. mellonella* model. This is an easy-to-handle and cost effective infection model to study *P. aeruginosa* pathogenicity, and a positive correlation has been observed between virulence of several *P. aeruginosa* mutants in *G. mellonella* and mice [32]. The *tolB* conditional mutant was strongly impaired in pathogenicity in *G. mellonella*, with an LD_90_ about 600,000 fold higher than that of the wild type (2.5 cells/larva and 1.5×10^6^ cells/larva for PAO1 and PAO1 Δ*tolB araC-*P_BAD_ *tolB*, respectively). In order to assess the effect of TolB depletion also on *P. aeruginosa* persistence *in vivo* during the infection, *G. mellonella* larvae were infected with a high infecting dose (corresponding to about 10^6^ CFUs) and the number of viable cells in the hemolymph was determined at 2 h post-infection. As shown in Fig. 3E, TolB-deficient cells displayed markedly reduced ability to persist in *G. mellonella* larvae with respect to wild-type or TolB-proficient mutant cells, while no significant differences in cell viability were observed between the same cell types in saline solution (Figure S3). This result indicates that TolB is also important for resistance to the antimicrobial defences of the *G. mellonella* hemolymph [32].

## Conclusions

In this work, we demonstrated that depletion of TolB, the periplasmic component of the Tol-Pal complex, abolishes *P. aeruginosa* growth *in vitro*, and markedly reduces persistence and pathogenicity in an animal infection model, as well as resistance to human serum and several antibiotics. This evidence leads us to propose TolB as a suitable candidate for the development of new drugs against *P. aeruginosa*. Since TolB is a soluble protein residing in the periplasmic space [1], [15], it should be more accessible to drugs than cytosolic targets, and drug binding to TolB could delay later extrusion by efflux pumps, which represent key components of both intrinsic and acquired resistance in *P. aeruginosa* [39]. Considering the high level of intrinsic antibiotic resistance in *P. aeruginosa* [40], [41], and the overall increase in drug susceptibility observed in TolB-depleted *P. aeruginosa* cells (Figs. 3D and 5; Table 2), a potential anti-TolB compound could also exhibit synergism with available antibiotics, likely revitalizing some of our current therapeutic options. It should be noted that, although not essential for growth *in vitro*, the Tol-Pal complex is important for antibiotic resistance and pathogenicity also in other Gram-negative pathogens (reviewed in [8]), suggesting that anti-TolB therapy could be ultimately beneficial for the treatment of different bacterial infections.

## Supporting Information

Figure S1 **SEM and TEM images (left and right panels, respectively) of TolB-deficient mutant cells grown as described in the legend to** **Figure 3A** **.** Bars: 1 µm (left panel) or 0.5 µm (right panel).
(PDF)

Figure S2 **Growth curves of the wild-type strain PAO1 (circles, solid lines) and the PAO1** ***tolB*** **conditional mutant (diamonds, dashed lines) in microtiter plates at 37°C in MH broth supplemented with increasing concentrations of sucrose (0–20%).** The graph is representative of three independent experiments giving similar results.
(PDF)

Figure S3 **Viability of PAO1 wild-type cells (WT), TolB-deficient mutant cells (** ***tolB*** **) and TolB-proficient mutant cells (** ***tolB*** **TolB^+^) after 3-h incubation in saline solution at 37°C, expressed as percent survival with respect to the number of viable cells at time 0.** Results are the mean (± SD) of four independent experiments. No significant differences were detected (one-way ANOVA).
(PDF)

Table S1 **Primers used in this study.**
(PDF)
