# Supplementary material for: The Periplasmic Protein TolB as a Potential Drug Target in Pseudomonas aeruginosa
Source: PLoS One. 2014 Aug 5;9(8):e103784. doi: 10.1371/journal.pone.0103784 (PMC4122361; doi:10.1371/journal.pone.0103784)
Supplement: Figure S1 — SEM and TEM images (left and right panels, respectively) of TolB-deficient mutant cells grown as described in the legend to Figure 3A . Bars: 1 µm (left panel) or 0.5 µm (right panel). (PDF) [file pone.0103784.s001.pdf]

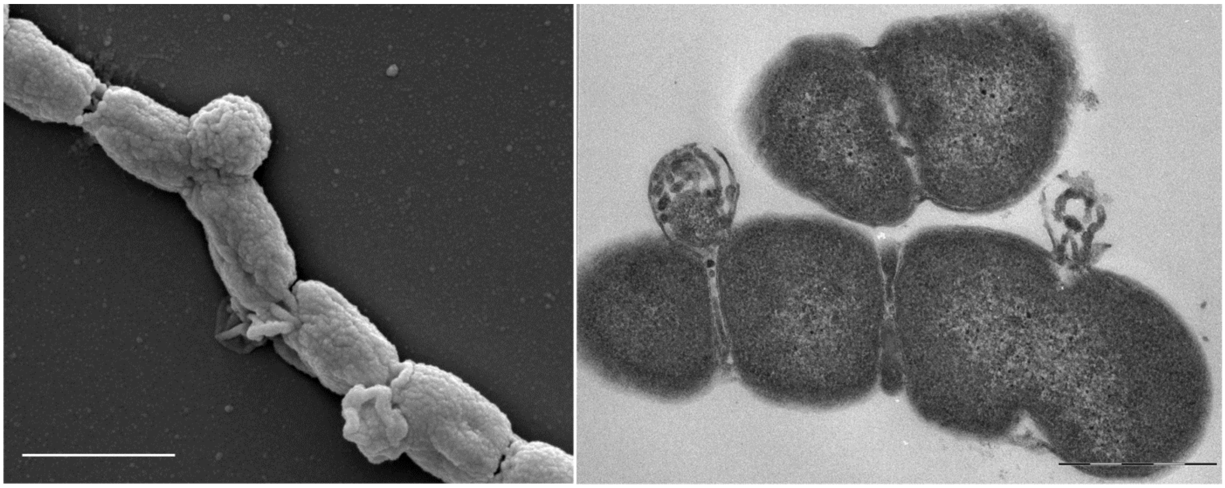

Figure S1. SEM and TEM images (left and right panels, respectively) of TolB-deficient mutant cells grown as described in the legend to Figure 3A. Bars: 1 µm (left panel) or 0.5 µm (right panel).
