# Supplementary material for: The Periplasmic Protein TolB as a Potential Drug Target in Pseudomonas aeruginosa
Source: PLoS One. 2014 Aug 5;9(8):e103784. doi: 10.1371/journal.pone.0103784 (PMC4122361; doi:10.1371/journal.pone.0103784)
Supplement: Figure S2 — Growth curves of the wild-type strain PAO1 (circles, solid lines) and the PAO1 tolB conditional mutant (diamonds, dashed lines) in microtiter plates at 37°C in MH broth supplemented with increasing concentrations of sucrose (0–20%). The graph is representative of three independent experiments giving similar results. (PDF) [file pone.0103784.s002.pdf]

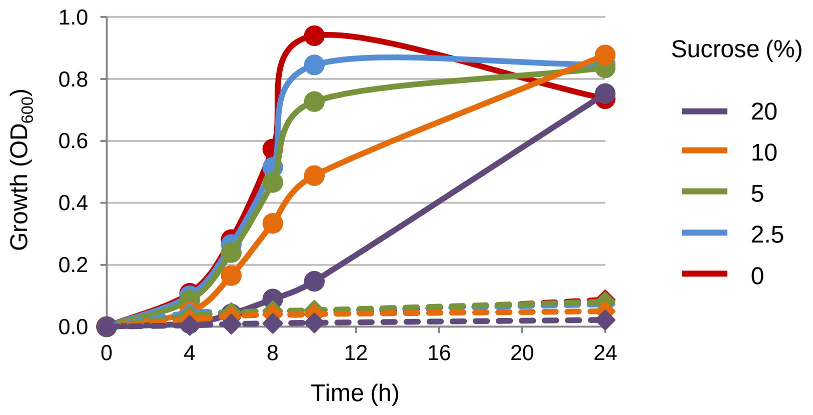

Figure S2. Growth curves of the wild-type strain PAO1 (circles, solid lines) and the PAO1 *tolB* conditional mutant (diamonds, dashed lines) in microtiter plates at 37°C in MH broth supplemented with increasing concentrations of sucrose (0-20%). The graph is representative of three independent experiments giving similar results.
