# Supplementary material for: The Periplasmic Protein TolB as a Potential Drug Target in Pseudomonas aeruginosa
Source: PLoS One. 2014 Aug 5;9(8):e103784. doi: 10.1371/journal.pone.0103784 (PMC4122361; doi:10.1371/journal.pone.0103784)
Supplement: Figure S3 — Viability of PAO1 wild-type cells (WT), TolB-deficient mutant cells ( tolB ) and TolB-proficient mutant cells ( tolB TolB+) after 3-h incubation in saline solution at 37°C, expressed as percent survival with respect to the number of viable cells at time 0. Results are the mean (± SD) of four independent experiments. No significant differences were detected (one-way ANOVA). (PDF) [file pone.0103784.s003.pdf]

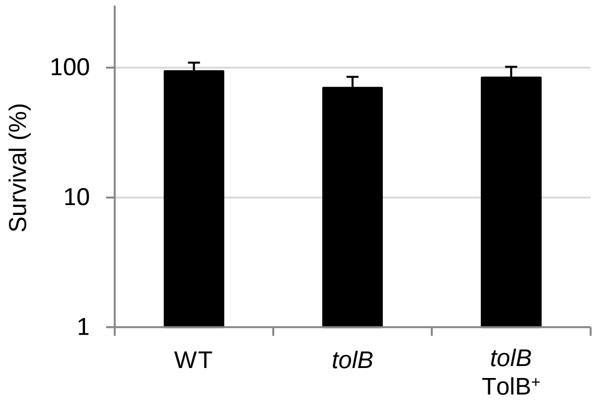

Figure S3. Viability of PAO1 wild-type cells (WT), TolB-deficient mutant cells (*tolB*) and TolB-proficient mutant cells (*tolB* $TolB^{+}$) after 3-h incubation in saline solution at 37°C, expressed as percent survival with respect to the number of viable cells at time 0. Results are the mean (± SD) of four independent experiments. No significant differences were detected (one-way ANOVA).
