# Supplementary material for: The Periplasmic Protein TolB as a Potential Drug Target in Pseudomonas aeruginosa
Source: PLoS One. 2014 Aug 5;9(8):e103784. doi: 10.1371/journal.pone.0103784 (PMC4122361; doi:10.1371/journal.pone.0103784)
Supplement: Table S1 — Primers used in this study. (PDF) [file pone.0103784.s004.pdf]

Table S1. Primers used in this study [a]

Primer name	Sequence [b]	Restriction site	Application
*tolB*_FW	5'-CCCAAGCTTCTGTGAGTACCCTGATTCGC-3'	HindIII	Generation of the pBEM9-*tolB* plasmid
*tolB*_RV	5'-CGGAATTCAGTTCAGGTAAGGGGACC-3'	EcoRI	Generation of the pBEM9-*tolB* plasmid
tolB mut_UP_FW	5'-CCGCTCGAGGCCAAGGCCGCCG-3'	XhoI	Generation of the pDM4Δ*tolB* plasmid
tolB mut_UP_RV	5'-CGGGATCCAGCGCAATGCGAATCAGG-3'	BamHI	Generation of the pDM4Δ*tolB* plasmid
tolB mut_DOWN_FW	5'-CGGGATCCCGCTCAGGGCGATGTGC-3'	BamHI	Generation of the pDM4Δ*tolB* plasmid
tolB mut_DOWN_RV	5'-GCTCTAGACACGCAGGTGCTTGGG-3'	XbaI	Generation of the pDM4Δ*tolB* plasmid

[a] All PCRs were performed using the genomic DNA of *P. aeruginosa* PAO1 as the template.
[b] The restriction site used for cloning is underlined in the primer sequence.
